# Pangenome databases improve host removal and mycobacteria classification from clinical metagenomic data

**DOI:** 10.1093/gigascience/giae010

**Published:** 2024-04-04

**Authors:** Michael B Hall, Lachlan J M Coin

**Affiliations:** Department of Microbiology and Immunology, Peter Doherty Institute for Infection and Immunity, The University of Melbourne, Melbourne, 3000 Victoria, Australia; Department of Microbiology and Immunology, Peter Doherty Institute for Infection and Immunity, The University of Melbourne, Melbourne, 3000 Victoria, Australia

**Keywords:** host removal, metagenomics, *Mycobacterium tuberculosis*, taxonomic classification, benchmark

## Abstract

**Background:**

Culture-free real-time sequencing of clinical metagenomic samples promises both rapid pathogen detection and antimicrobial resistance profiling. However, this approach introduces the risk of patient DNA leakage. To mitigate this risk, we need near-comprehensive removal of human DNA sequences at the point of sequencing, typically involving the use of resource-constrained devices. Existing benchmarks have largely focused on the use of standardized databases and largely ignored the computational requirements of depletion pipelines as well as the impact of human genome diversity.

**Results:**

We benchmarked host removal pipelines on simulated and artificial real Illumina and Nanopore metagenomic samples. We found that construction of a custom kraken database containing diverse human genomes results in the best balance of accuracy and computational resource usage. In addition, we benchmarked pipelines using kraken and minimap2 for taxonomic classification of *Mycobacterium* reads using standard and custom databases. With a database representative of the *Mycobacterium* genus, both tools obtained improved specificity and sensitivity, compared to the standard databases for classification of *Mycobacterium tuberculosis*. Computational efficiency of these custom databases was superior to most standard approaches, allowing them to be executed on a laptop device.

**Conclusions:**

Customized pangenome databases provide the best balance of accuracy and computational efficiency when compared to standard databases for the task of human read removal and *M. tuberculosis* read classification from metagenomic samples. Such databases allow for execution on a laptop, without sacrificing accuracy, an especially important consideration in low-resource settings. We make all customized databases and pipelines freely available.

## Introduction

*Mycobacterium tuberculosis* is the bacterium that causes tuberculosis, which is a leading cause of death globally [1]. Tuberculosis is an ancient airborne disease that predominantly affects the lungs [2]. Whole-genome sequencing (WGS) with Illumina and Oxford Nanopore Technologies (Nanopore) platforms is increasingly being used for *M. tuberculosis* diagnostic applications such as drug resistance prediction, lineage determination, and identification of putative transmission clusters [3–6]. Currently, most WGS applications for *M. tuberculosis* rely on culturing of the bacterium on either solid or liquid media, which can take days to weeks. Sequencing of *M. tuberculosis* directly from patient sample (sputum) is a much desired solution as it provides faster time to results and does not require the infrastructure necessary for culture.

A number of issues exist when sequencing *M. tuberculosis* direct from sputum—and, indeed, any human-associated metagenomic sample. *M. tuberculosis* genomic DNA is generally scarce in such metagenomic samples, with host and other bacterial DNA dominating [7, 8]. Capture of *M. tuberculosis* DNA can be attempted during sample preparation or computationally during sample quality control. We focus here on the computational extraction of *M. tuberculosis* DNA from metagenomic sputum samples. Previous work has shown that removal of non–*M. tuberculosis* sequencing reads from metagenomic samples is crucial for reducing false-positive and false-negative variant calls in downstream analyses [9], even for samples with low levels of contamination. One solution that is common in bioinformatic pipelines is contamination removal using either alignment tools or taxonomic classifiers. Kraken [10] is a popular taxonomic classifier used for this purpose, with standardized databases generally being used [9, 11]. Alignment to the *M. tuberculosis* H37Rv reference genome is another favored choice [12, 13], although this approach has been shown to still propagate false variant calls [9]. A more robust alignment approach is competitive mapping, where alignment databases are constructed to include common contaminants [14]. The idea with competitive mapping is that including a variety of species decreases the likelihood that reads from organisms with a similar sequence will incorrectly map to your organism of interest.

While *M. tuberculosis* contamination removal and read classification have been assessed previously [9, 15], these studies have focused on Illumina sequencing data. However, Nanopore data are becoming a popular choice for *M. tuberculosis* metagenomic studies [8, 16]. As Nanopore sequencing can be performed on a laptop, analysis pipelines should use computational resources that make them executable on such machines. Goig et al. [9] recommended use of the standard kraken database for removal of contamination and extrication of *M. tuberculosis* DNA, but this database uses upward of 65 GB of memory, an amount that is not available on (nearly all) laptops or indeed most desktop computers, thus requiring users to have access to high-performance computing resources.

In this study, we assess a variety of tools for the removal of human reads in metagenomic samples, as well as the classification of *M. tuberculosis* reads from both simulated and real Illumina and Nanopore sequencing platforms. We note that the removal of host reads is applicable to any human-associated metagenomic samples. We place a strong emphasis on computational resource usage in addition to accuracy. While we assess standard databases, we also create custom databases for both tasks and make them freely available with this work. This curation of custom databases allows us to keep computational resource usage low, while maintaining high(er) read classification accuracy.

## Results

We generated an *in silico* metagenomic readset from a variety of organisms (human, bacteria, virus) at ratios commonly seen in *M. tuberculosis* metagenomic samples (see “Generating *in silico* metagenomic reads”) [8]. After removing Nanopore reads shorter than 500 bp or with an ambiguous base, we were left with 234,984 reads with a total of 2.48 Gbp [17]. For Illumina, after removing reads with an ambiguous base, we retained 275,3282 read pairs with a combined total of 826 Mbp [18].

Additionally, we created an artificial real metagenomic dataset by combining human, *M. tuberculosis*, and mock metagenomic community (Zymo) data [19]. The reason for creating an artificial real dataset in this way is so that we can be highly confident of the true taxa of each read. While we cannot be certain of the exact taxa for each read in the mock community, no human or *Mycobacterium* DNA are present in this sample. We removed all Nanopore reads shorter than 500 bp and downsampled each component to a maximum of 3 Gbp for both sequencing modalities. We retained 1,057,160 Nanopore reads and 31,172,963 Illumina read pairs for a total of 5.64 Gbp and 7.68 Gbp, respectively.

### Removal of human reads

A primary focus of this work is capturing *M. tuberculosis* reads from metagenomic samples, with a secondary aim of doing this in low-resource computational settings (e.g., on a laptop). In general, for these types of samples, human reads are undesirable and so their removal is an important first step. While human read removal could be built into the broader classification of such a sample, removal *ab initio* reduces memory requirements (smaller, modular databases can be used), decreases file sizes and therefore runtimes in downstream analyses, and avoids accidental patient DNA ending up in data submitted to public archives. In addition, host read removal is a task common to most metagenomic applications, so the separate assessment is likely to be of interest to a wider audience.

We benchmarked 6 configurations from both *k*-mer– and alignment-based approaches for classifying reads as being human or not (see “Human read removal”). The *k*-mer–based methods include the human read removal tool (HRRT) [20] and kraken (RRID:SCR_005484) [10] with the default human database [21] and a database we built from the 97 assemblies used by the Human Pangenome Reference Consortium (HPRC) [22, 23]. The alignment-based approaches include minimap2 (RRID:SCR_018550) [24], Hostile [25] (which uses minimap2 for Nanopore and Bowtie2 [26] for Illumina), and minimap2 followed by winnowmap [27] (*miniwinnow*; note, this option was not applicable for Illumina data). The reference used for each alignment method was the CHM13v2 assembly [28] (NCBI RefSeq accession GCF_009914755.1) plus human leukocyte antigen sequences [25]. Note that the simulated and artificial real human reads are from genomes that are not present in any of the human removal databases used here.

#### Simulated Nanopore

Table 1 presents the computational and accuracy performance for each method on the simulated Nanopore data (see Supplementary Table S1 for full counts). From this, we see that all methods have very high sensitivity and specificity. All methods, except HRRT, produce an equally good balance of sensitivity and specificity, with a Youden’s index of 0.9998. Kraken (both databases) was an order of magnitude faster than HRRT, Hostile, and miniwinnow and at least 4 times faster than minimap2. Lastly, the *k*-mer–based classification methods had much lower peak memory usage than their alignment-based competitors, with memory usage low enough to be suitable for operation on most laptop devices (<8 GB).

**Table 1:** Performance of human read classification—simulated Nanopore

Method	Rate (reads/s)*	Memory (GB)^†^	Specificity (95% CI)	Sensitivity (95% CI)	Youden’s index (95% CI)
HRRT	281	**1.0**	**1.0** (0.9999–1.0)	0.9809 (0.9802–0.9817)	0.9809 (0.9801–0.9817)
Hostile	263	12.8	**1.0** (0.9999–1.0)	0.9998 (0.9998–0.9999)	**0.9998** (0.9997–0.9999)
minimap2	412	9.0	0.9999 (0.9999–1.0)	0.9998 (0.9998–0.9999)	**0.9998** (0.9996–0.9999)
miniwinnow	278^‡^	9.0^§^	0.9999 (0.9998–1.0)	**0.9999** (0.9998–0.9999)	**0.9998** (0.9997–0.9999)
kraken default	1,618	4.1	**1.0** (0.9999–1.0)	0.9998 (0.9997–0.9999)	**0.9998** (0.9996–0.9999)
kraken HPRC	**2,384**	4.7	0.9999 (0.9999–1.0)	0.9998 (0.9997–0.9999)	**0.9998** (0.9996–0.9998)

Bold text indicates the best performing method for that metric. CI, confidence interval (Wilson score interval); HPRC, Human Pangenome Reference Consortium; HRRT, human read removal tool.

*Average from 10 executions.

^†^Maximum from 10 executions.

^‡^As winnowmap is run on the minimap2 output, the runtime is the summation of the 2 processes.

^§^As winnowmap is run on the minimap2 output, the maximum memory is the higher of the 2 processes, which was minimap2. The winnowmap peak memory was 3.0 GB.

Importantly, kraken did not classify any *Mycobacterium* reads as being human. However, all other methods did, albeit a very small number, with miniwinnow having the most, with 2 *M. tuberculosis* and 3 *Mycobacterium kansasii* reads being called as human.

#### Real Nanopore

Table 2 shows the computational performance and accuracy metrics for human read removal from the artificial real Nanopore data (see Supplementary Table S2 for full counts). The main difference from the simulated Nanopore data is that the alignment-based methods (Hostile, minimap2, and miniwinnow) provide a higher Youden’s index (0.9844) to the *k*-mer–based approaches (with no confidence interval overlap). Sensitivity and specificity are lower than for simulated data, but by a very small amount. Kraken is again an order of magnitude faster than other methods, and *k*-mer–based approaches have laptop-compatible memory usage.

**Table 2:** Performance of human read classification—real Nanopore

Method	Rate (reads/s)*	Memory (GB)^†^	Specificity (95% CI)	Sensitivity (95% CI)	Youden’s index (95% CI)
HRRT	488	**1.1**	0.9969 (0.9968–0.997)	0.9774 (0.9766–0.9781)	0.9742 (0.9733–0.9751)
Hostile	333	14.3	**0.9986** (0.9985–0.9987)	0.9858 (0.9852–0.9864)	**0.9844** (0.9837–0.9851)
minimap2	405	11.8	0.9985 (0.9984–0.9986)	0.986 (0.9853–0.9866)	**0.9844** (0.9837–0.9851)
miniwinnow	282^‡^	11.8^§^	0.9968 (0.9967–0.997)	**0.9876** (0.987–0.9882)	**0.9844** (0.9837–0.9851)
kraken default	4,322	4.1	0.9973 (0.9972–0.9974)	0.9812 (0.9804–0.9818)	0.9784 (0.9776–0.9792)
kraken HPRC	**8,081**	4.8	0.9962 (0.9961–0.9963)	0.983 (0.9823–0.9837)	0.9792 (0.9784–0.98)

Bold text indicates the best performing method for that metric. CI, confidence interval (Wilson score interval); HPRC, Human Pangenome Reference Consortium; HRRT, human read removal tool.

*Average from 10 executions.

^†^Maximum from 10 executions.

^‡^As winnowmap is run on the minimap2 output, the runtime is the summation of the 2 processes.

§ As winnowmap is run on the minimap2 output, the maximum memory is the higher of the 2 processes, which was minimap2. The winnowmap peak memory was 4.5 GB.

Each method classified a number of *M. tuberculosis* reads as being human, with HRRT classifying the most with 1,657 (1.6% of total *M. tuberculosis* reads) and Hostile the least with 147 (0.1%).

#### Simulated Illumina

Table 3 presents the results for human read removal on simulated Illumina data (see Supplementary Table S3 for full counts). The simulated Illumina results have lower sensitivity than Nanopore, with minimap2 providing the highest sensitivity (0.9837). All methods performed exceptionally for specificity, with HRRT producing no false positives (FPs) and minimap2 producing the highest Youden’s index (0.9836). The runtime comparison of the methods on Illumina data is much the same as for Nanopore data, with kraken being an order of magnitude faster than the other methods. Aside from minimap2, peak memory usage for all methods is low enough to be suitable for operation on most laptop devices (<8 GB).

**Table 3:** Performance of human read classification—simulated Illumina

Method	Rate (reads/s)*	Memory (GB)^†^	Specificity (95% CI)	Sensitivity (95% CI)	Youden’s index (95% CI)
HRRT	15,427	**1.0**	**1.0** (1.0–1.0)	0.9017 (0.9013–0.902)	0.9017 (0.9013–0.902)
Hostile	17,302	4.1	**1.0** (1.0–1.0)	0.981 (0.9808–0.9811)	0.981 (0.9808–0.9811)
minimap2	14,294	11.7	**1.0** (0.9999–1.0)	**0.9837** (0.9835–0.9838)	**0.9836** (0.9835–0.9838)
kraken default	**196,281**	4.1	**1.0** (1.0–1.0)	0.9737 (0.9736–0.9739)	0.9737 (0.9735–0.9739)
kraken HPRC	151,811	4.8	**1.0** (1.0–1.0)	0.9787 (0.9785–0.9789)	0.9787 (0.9785–0.9789)

Bold text indicates the best performing method for that metric. CI, confidence interval (Wilson score interval); HPRC, Human Pangenome Reference Consortium; HRRT, human read removal tool.

*Average from 10 executions.

^†^Maximum from 10 executions.

Minimap2 was the only method that classified *Mycobacterium* Illumina reads as being human: 11 *M. tuberculosis*, 1 *Mycobacterium ulcerans*, and 1 *M. kansasii*. In addition to those 13 *Mycobacterium* reads, the most common genera erroneously classified as human by minimap2 were *Xanthomonas* (*n* = 7) and *Streptococcus, Clostridium, Clostridioides*, and *Acinetobacter* (all *n* = 5). All 4 of the Hostile FPs were from human endogenous retrovirus K (HERV-K). Four of the FPs from both kraken databases were also HERV-K, along with 6 Epstein–Barr virus reads and 4 *Campylobacter* against the HPRC database.

Almost all of the missed human reads (false negatives [FNs]) for all methods were from unplaced scaffolds in the KOREF_S1v2.1 genome. We investigated whether this could be the result of contamination from nonhuman reads in the assembly. The KOREF genome was split into 100-bp overlapping pseudo-reads every 50 bp using the SeqKit (v2.4.0) command sliding [29]. The pseudo-reads were then classified with kraken using the standard database (see “Classification of *Mycobacterium* reads”). Of the 57,943,303 pseudo-reads, only 56 were classified as bacteria, with no family being overrepresented. As such, we can conclude these unplaced scaffolds are unlikely due to contamination.

#### Real Illumina

Table 4 shows the computational and accuracy performance of the human read removal methods on artificial real Illumina data (see Supplementary Table S4 for full counts). Interestingly, in contrast to the simulated Illumina data, kraken, with the human pangenome database (HPRC), had the highest Youden’s index (0.9952) and sensitivity (0.9959). HRRT and Hostile had the highest specificity (0.9999). Computational performance was the same as with the simulated Illumina data.

**Table 4:** Performance of human read classification—real Illumina

Method	Rate (reads/s)*	Memory (GB)^†^	Specificity (95% CI)	Sensitivity (95% CI)	Youden’s index (95% CI)
HRRT	25,857	**1.5**	**0.9999** (0.9999–0.9999)	0.9718 (0.9718–0.9719)	0.9717 (0.9716–0.9718)
Hostile	33,967	7.3	**0.9999** (0.9999–0.9999)	0.9934 (0.9933–0.9934)	0.9933 (0.9933–0.9933)
minimap2	35,105	11.6	0.9996 (0.9996–0.9996)	0.9911 (0.991–0.9911)	0.9907 (0.9906–0.9907)
kraken default	**402,528**	4.2	0.9997 (0.9997–0.9998)	0.9937 (0.9936–0.9937)	0.9934 (0.9934–0.9935)
kraken HPRC	241,234	4.8	0.9993 (0.9993–0.9993)	**0.9959** (0.9959–0.996)	**0.9952** (0.9952–0.9953)

Bold text indicates the best performing method for that metric. CI, confidence interval (Wilson score interval); HPRC, Human Pangenome Reference Consortium; HRRT, human read removal tool.

*Average from 10 executions.

^†^Maximum from 10 executions.

Each method classified a small amount of *M. tuberculosis* reads as being human, with minimap2 classifying the most with 2,476 (0.01% of total *M. tuberculosis* reads) and HRRT the least with 84 (0.0004%).

### Classification of *Mycobacterium* reads

Having assessed the removal of human reads from simulated and real metagenomic samples, we turn to read classification. In particular, we focus on being able to classify *M. tuberculosis* reads to the correct species, with as few false positives and false negatives as possible. For this section of the analysis, we use the nonhuman reads. For the simulated dataset, we are left with 100,733 Nanopore reads (460 Mbp) and 1,417,015 Illumina read pairs (424 Mbp). For the real dataset, we have 915,209 Nanopore reads (3.47 Gbp) and 21,172,961 Illumina read pairs (4.68 Gbp).

We benchmarked kraken and minimap2 for read classification, using 3 different databases for each tool. For kraken, we used the standard database (standard) and a version of the standard database that is capped at 8 GB (standard-8). In addition, we created a *Mycobacterium*-specific database (*Myco*), which contains a RefSeq genome from each species in the *Mycobacteriaceae* family plus a variety of other genera (see *Mycobacterium* read classification) [30]. For minimap2, we created an *M. tuberculosis*–specific database (*MTB*) using the *M. tuberculosis* H37Rv reference genome and 17 high-quality *M. tuberculosis* genomes from lineages 1–6 [31, 32]. We also created a *Mycobacterium*-specific database (*Myco*) with a RefSeq genome from each species in the *Mycobacterium* genus [33]. And lastly, we used the decontamination database from the Clockwork pipeline [14] but also added the 17 high-quality *M. tuberculosis* genomes [34]. This Clockwork database contains many expected contaminants in a sputum sample such as nasopharyngeal flora, human immunodeficiency virus, and the human genome [14].

#### Simulated Nanopore

Table 5 shows the *M. tuberculosis* read classification results for simulated Nanopore data (see Supplementary Table S5 for full counts). From this we see that minimap2 with the Clockwork and *Myco* databases had the highest sensitivity (1.0) with 1 and 2 false-negative call(s), respectively. Kraken (all databases) had the highest specificity (1.0), with only 1 FP for the *Myco* database and none for the standard. Minimap2 with the *Myco* database gave the highest Youden’s index of 0.9999, producing only 2 FNs and 3 FPs. Kraken with the standard-8 database was the fastest method (4,884 reads per second). Memory consumption varied quite a bit across methods, with minimap2’s MTB database using the lowest at 2.0 GB. Notably, kraken standard would not be executable on a computer with 16 GB of memory, while minimap2 Clockwork is very close to this limit.

**Table 5:** Performance of *M. tuberculosis* read classification—simulated Nanopore

Method	Rate (reads/s)*	Memory (GB)^†^	Specificity (95% CI)	Sensitivity (95% CI)	Youden’s index (95% CI)
kraken standard	1,406	66.8	**1.0** (0.9999–1.0)	0.7408 (0.7373–0.7443)	0.7408 (0.7371–0.7443)
kraken standard-8	**4,884**	7.8	**1.0** (0.9999–1.0)	0.4196 (0.4157–0.4236)	0.4196 (0.4155–0.4236)
kraken *Myco*	4,286	8.2	**1.0** (0.9998–1.0)	0.9953 (0.9947–0.9958)	0.9953 (0.9945–0.9958)
minimap2 Clockwork	349	14.0	0.9983 (0.9978–0.9987)	**1.0** (0.9999–1.0)	0.9983 (0.9977–0.9987)
minimap2 MTB	418	**2.0**	0.9735 (0.9715–0.9753)	0.9703 (0.969–0.9717)	0.9438 (0.9405–0.9469)
minimap2 *Myco*	368	7.9	0.9999 (0.9997–1.0)	**1.0** (0.9999–1.0)	**0.9999** (0.9996–1.0)

Bold text indicates the best performing method for that metric. CI, confidence interval (Wilson score interval).

*Average from 10 executions.

^†^Maximum from 10 executions.

#### Real Nanopore

Table 6 presents the *M. tuberculosis* classification results for the artificial real Nanopore dataset (see Supplementary Table S6 for full counts). These results show much the same as the simulated data, with minimap2 and the *Myco* database providing the highest Youden’s index (0.9806) and sensitivity (0.9811), although the confidence intervals for these metrics do overlap with minimap2 and the Clockwork database. Kraken (all databases) gave no FPs and thus had a specificity of 1.0. The computational results gave similar results to the simulated data, with kraken’s standard-8 database being the fastest and minimap2 with the MTB database having the lowest memory footprint.

**Table 6:** Performance of *M. tuberculosis* read classification—real Nanopore

Method	Rate (reads/s)*	Memory (GB)^†^	Specificity (95% CI)	Sensitivity (95% CI)	Youden’s index (95% CI)
kraken standard	6735	67.0	**1.0** (1.0–1.0)	0.7114 (0.7086–0.7142)	0.7114 (0.7086–0.7142)
kraken standard-8	**14,828**	7.7	**1.0** (1.0–1.0)	0.4479 (0.4449–0.451)	0.4479 (0.4448–0.451)
kraken *Myco*	6,755	8.2	**1.0** (1.0–1.0)	0.9773 (0.9764–0.9782)	0.9773 (0.9764–0.9782)
minimap2 Clockwork	1,225	13.8	0.9995 (0.9995–0.9996)	0.9808 (0.9799–0.9816)	0.9803 (0.9794–0.9812)
minimap2 MTB	1,892	**2.5**	0.9819 (0.9816–0.9821)	0.945 (0.9436–0.9464)	0.9269 (0.9252–0.9285)
minimap2 *Myco*	332	7.9	0.9995 (0.9995–0.9995)	**0.9811** (0.9803–0.9819)	**0.9806** (0.9797–0.9815)

Bold text indicates the best performing method for that metric. CI, confidence interval (Wilson score interval).

*Average from 10 executions.

^†^Maximum from 10 executions.

#### Simulated Illumina

Table 7 presents the *M. tuberculosis* read classification results for simulated Illumina data (see Supplementary Table S7 for full counts). The best performers were minimap2 Clockwork with the highest sensitivity (0.9999) and Youden’s index (0.9998) and kraken (all databases) and minimap2 (*Myco* database) the highest specificity (1.0). In terms of runtime, kraken standard-8 was again the fastest, but all methods ran in under 1.5 minutes. Memory consumption was again similar to Nanopore data, with minimap2 MTB using the lowest (0.7 GB) and minimap2 Clockwork and kraken standard using more than 16 GB.

**Table 7:** Performance of *M. tuberculosis* read classification—simulated Illumina

Method	Rate (reads/s)*	Memory (GB)^†^	Specificity (95% CI)	Sensitivity (95% CI)	Youden’s index (95% CI)
kraken standard	51,079	66.8	**1.0** (1.0–1.0)	0.1634 (0.1622–0.1647)	0.1634 (0.1622–0.1647)
kraken standard-8	193,794	7.7	**1.0** (1.0–1.0)	0.0548 (0.0541–0.0556)	0.0548 (0.0541–0.0556)
kraken *Myco*	**194,893**	8.2	**1.0** (1.0–1.0)	0.9731 (0.9726–0.9737)	0.9731 (0.9726–0.9737)
minimap2 Clockwork	29,100	20.7	0.9999 (0.9999–0.9999)	**0.9999** (0.9999–1.0)	**0.9998** (0.9998–0.9999)
minimap2 MTB	91,734	**0.7**	0.9991 (0.999–0.9991)	0.9475 (0.9467–0.9482)	0.9465 (0.9457–0.9473)
minimap2 *Myco*	49,727	11.0	**1.0** (1.0–1.0)	0.9996 (0.9995–0.9996)	0.9995 (0.9994–0.9996)

Bold text indicates the best performing method for that metric. CI, confidence interval (Wilson score interval).

*Average from 10 executions.

^†^Maximum from 10 executions.

Minimap2 with the *Myco* database made 148 FNs and 56 FPs. Of these, all FNs were aligned to non–*M. tuberculosis* species, with 50 being assigned to *Mycobacterium angelicum* and 36 to *Mycobacterium shinjukuense*. No genera were overrepresented in the 56 FPs. For the Clockwork database, there were 24 FNs and 204 FPs. Eight of the FNs were aligned to *Mycobacterium*, with the remainder being unmapped, while there were no overrepresented genera in the FPs.

#### Real Illumina

Table 8 shows the result for *M. tuberculosis* read classification on the artificial real Illumina data (see Supplementary Table S8 for full counts). We see the same trends as with the simulated data, with minimap2 and the Clockwork database providing the highest Youden’s index (0.9956) and sensitivity (0.9960) and kraken (all databases) the highest specificity (1.0). Kraken was again the fastest method (647,608 reads per second) and minimap2 with the MTB database the lowest memory usage (2.0 GB).

**Table 8:** Performance of *M. tuberculosis* read classification—real Illumina

Method	Rate (reads/s)*	Memory (GB)^†^	Specificity (95% CI)	Sensitivity (95% CI)	Youden’s index (95% CI)
kraken standard	239,420	67.2	**1.0** (1.0–1.0)	0.0731 (0.073–0.0732)	0.0731 (0.073–0.0732)
kraken standard-8	**647,608**	7.8	**1.0** (1.0–1.0)	0.0146 (0.0146–0.0147)	0.0146 (0.0146–0.0147)
kraken *Myco*	398,597	8.3	**1.0** (1.0–1.0)	0.9715 (0.9714–0.9716)	0.9715 (0.9714–0.9716)
minimap2 Clockwork	56,485	22.2	0.9996 (0.9996–0.9996)	**0.996** (0.996–0.9961)	**0.9956** (0.9956–0.9956)
minimap2 MTB	82,194	**2.0**	0.9884 (0.9883–0.9884)	0.9416 (0.9415–0.9417)	0.93 (0.9298–0.9301)
minimap2 *Myco*	11,066	12.9	0.9997 (0.9996–0.9997)	0.9955 (0.9954–0.9955)	0.9951 (0.9951–0.9951)

Bold text indicates the best performing method for that metric. CI, confidence interval (Wilson score interval).

*Average from 10 executions.

^†^Maximum from 10 executions.

Of the 89,330 FNs for minimap2’s Clockwork database, most were unmapped (62,826), but those that did map were aligned to *Mycobacterium* genomes, with 22 FNs mapping to the human genome.

Kraken, with the full-size and smaller standard databases, had much lower sensitivity than the other approaches for both sequencing technologies for *M. tuberculosis* read classification. This was predominantly due to it not being able to place the missed reads (FNs) at the species rank. In all cases, at least 90% of the FNs were correctly classified at the genus level, with the remainder not being placed even at the genus level.

### *M. tuberculosis* coverage analysis

In addition to looking at how many reads can be correctly classified as *M. tuberculosis*, we also investigated the breadth and depth of coverage of those retained reads. This is an important extra step that looks beyond how many reads are kept and identifies if the retained reads are evenly dispersed across the genome or concentrated in specific regions.

For both the simulated and real Nanopore data, we see effectively no drop in breadth of coverage after *M. tuberculosis* read classification (Supplementary Tables S9 and S10, respectively). This is despite the 2 standard kraken databases having low sensitivity and thus up to 50% drop in depth of coverage. The reason for this is due to the read length of Nanopore data. Even if kraken cannot classify all reads at the species level or does not have minimizers covering the whole *M. tuberculosis* genome (i.e., the standard-8 database), the reads are long enough that even a reduced number of reads and minimizer hits can lead to reads tiling the whole genome.

The simulated Illumina data give somewhat different results (Supplementary Table S11). We see that for kraken with the full and 8 GB standard database, we get a 40% and 80% reduction in breadth of coverage, respectively. This is due to both the low sensitivity, and thus drastically reduced depth, and the shorter read length of Illumina preventing tiling across minimizer gaps and depth gaps. The reduction in breadth and depth of coverage is not as severe for the standard kraken databases on the real Illumina data (Supplementary Table S12). In both the real and simulated Illumina datasets, all other method and databases did not see a noticeable reduction in breadth and depth of coverage, as was expected given the high sensitivity of those approaches.

## Discussion

We have performed a thorough evaluation of both human and *M. tuberculosis* read classification from simulated and artificial real metagenomic mixtures with both Nanopore and Illumina sequencing data. We have focused on human and *M. tuberculosis* classification in separate steps for 2 reasons. First, human reads are never required in typical *M. tuberculosis* analysis and are almost never uploaded to public sequence archives due to legal and ethical reasons. Second, removing human reads from the outset reduces computational time and memory usage in subsequent analyses and reduces the required bandwidth if data are to be uploaded to a public archive or cloud computing platform. As Nanopore sequencing can be performed on a laptop, the ability to perform contamination removal and read classification on such computers is an important consideration—especially with regards to memory consumption.

Most *M. tuberculosis* WGS work is done from culture and therefore does not contain much contamination by design. However, the eventual goal for the community is to move toward WGS direct-from-sputum analysis. Taking such samples directly from the patient, without a culturing step, will speed up time to results dramatically but does result in increased levels of contamination from the patient and other commensal species. Reducing the number of reads falsely classified as *M. tuberculosis* is important for downstream analyses. In particular, false non–*M. tuberculosis* reads can cause false positives and negatives during variant calling [9, 11]. As variant calling underpins many vital *M. tuberculosis* applications, such as transmission cluster detection and drug resistance prediction [4], host removal and read classification accuracy are critical.

We have tested standardized host removal methods and created a novel, custom kraken database from a diverse range of human genomes produced as part of the HPRC [22] (see “Data Availability”). We find that using this HPRC kraken database generally strikes the best balance between computational speed, memory consumption, and reduced false classifications across both Nanopore and Illumina data. However, if trying to reduce false positives, Hostile may be a slightly better solution—albeit slower than kraken and with higher memory usage. Another point of note is that while the sensitivity and specificity confidence intervals were often nonoverlapping for the best-performing methods, with the exception of HRRT, they were generally within <1% of other approaches. Therefore, we would not suggest changing existing pipelines that use any of these methods (aside from HRRT), unless improved computational performance is needed. While this analysis was applied in the context of *M. tuberculosis* metagenomic samples, removal of human data is not limited to such cases, and the methods tested here can be used in other metagenomic situations.

We note that the results from the artificial real data were generally 1% to 2% lower than the simulated data for Nanopore. This is likely explained by the fact that the real *M. tuberculosis* reads were from Nanopore R10.3, rather than the R10.4 that was used for simulated and other real datasets. R10.3 has been shown to have lower accuracy than R10.4 [35]. For Illumina, the inverse was true, with the real data having 1% to 2% better accuracy metrics. This is likely explained by the fact that the simulated reads were from a MiSeq platform, while most of the real reads were from a NovaSeq or HiSeq platform, which have a lower error rate than MiSeq [36]. Additionally, the real human reads, which were from the NovaSeq machine, have a read length of 250 bp compared to the 150 bp for the MiSeq (and HiSeq). Having longer, more accurate reads will inevitably improve the ability to correctly assign a read to the true taxon.

When comparing human read removal between the 2 sequencing technologies, we found real Nanopore data resulted in  0.5% lower sensitivity across the different pipelines and databases tested, but specificity was much the same.

For *M. tuberculosis* read classification, we tested some standard databases for minimap2 and kraken and also constructed customized *M. tuberculosis* databases (see “Data Availability”). There is no clear best performer across all metrics. If computational considerations are more important, kraken with the *Mycobacterium* representative database would be a good choice. However, for accuracy, the *Myco* and Clockwork minimap2 databases are our 2 recommendations. The choice of which of these databases to use will be up to individual users and their computational resource availability and whether reduction of false positives or negatives is of more importance.

In addition to looking at how many reads were correctly classified as *M. tuberculosis*, we also investigated any breadth and depth of genome coverage changes given the retained reads. Unsurprisingly, the methods with high sensitivity did not have a noticeable reduction in either form of coverage. However, while kraken with the full and 8 GB standard database has much lower sensitivity than the other methods, only the Illumina data saw a reduction on breadth of coverage. This highlights one advantage of long reads over short reads: the ability to still span the whole genome, even with a large drop in depth.

An important finding to note from the *M. tuberculosis* classification work is that the *MTB* minimap2 database and the standard kraken database consistently gave inferior accuracy (Youden’s index) compared to the other pangenome databases used. This finding challenges the prevailing methods of aligning reads to the H37Rv reference or employing the standard kraken database for *M. tuberculosis* read classification [37, 38], thereby paving the way for improved accuracy and effectiveness in tuberculosis research and diagnostics with pangenome databases.

Although the read classification and custom databases were targeted at *M. tuberculosis*, this work should act as a guide for how to design such taxon-specific databases for other species. We advise beginning with simulations of metagenomic samples and determining how much diversity, and which rank, is required from your taxa of interest based on sensitivity results. In addition, false-positive classifications are a good guide for which taxa outside your species of interest may need to be included as a kind of bait.

In conclusion, we found that construction of custom human and *M. tuberculosis* databases can improve human read removal and *M. tuberculosis* read classification in simulated and real metagenomic samples. We make all custom databases freely available, along with usage examples (see “Data Availability”).

## Methods

### Generating *in silico* metagenomic reads

We simulated metagenomic Nanopore and Illumina sequencing reads to a mixture ratio that approximates that found in patient sputa [8], albeit with a slightly higher mycobacterial component. In total, 4.5 and 0.9 gigabases were generated for Nanopore and Illumina, respectively, at the following proportions: 46% each for bacteria and human, 6% *M. tuberculosis* complex (MTBC), and 1% each for virus and nontuberculous mycobacteria (NTM).

The reference genomes that reads were simulated from for these groups were gathered as follows. The references for the virus group were obtained using kraken’s (v2.1.2) [10] --download-library functionality. The viral library was downloaded on 15 June 2023. The human genome from which the reads were simulated was the Korean reference KOREF_S1v2.1 (RefSeq accession GCA_020497085.1) [39], with contigs shorter than 10 kbp removed. The bacterial references were obtained by first downloading the bacteria library through kraken, followed by a subsampling due to the size (166 Gb) of the resulting FASTA file. We subsampled the file by first removing sequences with a length <50 kbp. We then extracted each sequence into its own FASTA file under a directory for the genus of the sequence—excluding the *Mycobacterium* genus [40, 41]. Genera were randomly subsampled to contain a maximum of 1,000 assemblies. Each genus was then reduced to a representative subset using Assembly Dereplicator (commit 2dfcb14) [42] by keeping only 10% of the assemblies for each genus (-f 0.1). The NTM references selected were *Mycobacterium abscessus* (accession GCF_017190695.1), *Mycobacterium avium* (GCF_020735285.1), *M. kansasii* (GCA_014701265.1), *M. ulcerans* (GCF_000013925.1), *Mycobacterium intracellulare* (GCF_016756075.1), *Mycobacterium terrae* (GCF_010727125.1), and *Mycobacterium fortuitum* (GCF_001307545.1). The MTBC reference is a lineage 1 assembly (GCF_932530395.1).

We used Badreads (v0.4.0) [43] to produce the simulated Nanopore reads for each group, specifying the number of bases in the appropriate proportions mentioned above. For all groups, we specified no junk or random reads and 0.5% chimeric reads. In addition, for the MTBC, virus, and NTM groups, we used a nondefault length option --length 4000,3000 to produce reads with a mean length 4,000 bp and a standard deviation of 3,000. Defaults were used for all other options (the default error model is trained on real R10.4.1 Nanopore reads from 2023 with median error rate 6.3%).

Illumina reads were simulated with ART (v2016.06.05) [44], using a similar approach as for Nanopore to generate reads for each group. We simulated paired reads from a MiSeq v3 system (-ss MSv3) with a read length of 150, a mean fragment length of 250, and fragment length standard deviation 10 (-l 150 -m 250 -s 10). This system has an error rate of 0.1% [45].

We filtered the simulated Nanopore reads to remove any read with a length <500 bp or an ambiguous nucleotide (non-ACGT) and removed simulated Illumina reads with any ambiguous base.

### Creation of an artificial real metagenomic dataset

We created Nanopore and Illumina metagenomic datasets by combining real sequencing reads into an artificial metagenomic dataset. By doing this, we can be highly confident of the true taxon for each read in the dataset.

We used samples that have matched Illumina and Nanopore sequencing to ensure that differences are purely driven by technological differences and not composition differences. For the human component, we combined reads from 3 individuals in the 1000 Genomes Project [46]. Nanopore data for the human component were downloaded from the 1000G ONT Sequencing Consortium [47]: HG00277 Finnish Male with Illumina NovaSeq 6000 (accession: ERR3241786) and Nanopore R10.4 [48]; NA19318 Luhya, Kenya Male with Illumina NovaSeq 6000 (accession: ERR3239713) and Nanopore R10.4 [49] (basecalled with Dorado v0.3.4); HG03611 Bengali, Bangladesh Female with Illumina NovaSeq 6000 (accession: ERR3243073); and Nanopore R10.4 [50] (basecalled with Dorado v0.3.4). Each human readset was randomly downsampled to 1 Gbp using rasusa (v0.7.1) [51]. For the *M. tuberculosis* component, we used Illumina HiSeq 4000 (accession: ERR245682) and Nanopore R10.3 (accession: ERR8170871) [52] (we used R10.3 as there were no R10.4 *M. tuberculosis* WGS datasets publicly available). For the bacterial component, we used Illumina MiSeq (accession: ERR7255689) and Nanopore R10.4 (accession: ERR7287988) reads from the ZymoBIOMICS HMW DNA Standard D6322 (Zymo Research), which contains 7 bacterial and 1 fungal strain(s) [53]—none of which are *Mycobacterium*. We removed Nanopore reads from all datasets with a length less than 500 bp, and the *M. tuberculosis* and Zymo datasets were downsampled to 3 Gbp with rasusa. All human, *M. tuberculosis*, and Zymo reads were combined into a single artificial metagenome file.

### Human read removal

We tested 6 configurations for removal of human reads from our simulated Nanopore metagenomic dataset: the human read removal tool (HRRT; v2.1.0) [20], with the -x -r options to remove human reads instead of masking them and write them to a separate file; minimap2 (v2.26) [24], with the -x map-ont option; Hostile (v0.0.3) [25], using the minimap2 aligner option; the fourth configuration by running winnowmap (v2.0) [27] on the nonhuman reads from the minimap2 configuration mentioned previously, using the same options as minimap2 (we label this configuration *miniwinnow*); and the remaining 2 configurations using kraken with 2 different databases. One was built using only the default human library that comes with the --download-library human option. This library contains the CHM13v2 [28] and GRCh38 references—we term this configuration *kraken default*. The second database consists of all 97 assemblies from the Human Pangenome Reference Consortium [22]—we call this database *kraken HPRC*.

It should be noted that while Hostile is using minimap2 and aligning to the same reference genome as the minimap2 configuration, it uses base-level alignment to a SAM file (-a), while the minimap2 configuration uses *approximate* mapping (default), which is generally less accurate but faster [24].

For the Illumina dataset, we used 5 configurations—the same methods as above, minus the miniwinnow approach as it only works for long reads. Hostile uses Bowtie2 [26] as the read aligner when operating on Illumina data. For Illumina, we used the -x sr option in minimap2 instead, and in kraken, we additionally used the --paired option.

We assess the performance of these configurations using sensitivity, specificity, Youden’s index, peak memory usage, and runtime (rate—reads per second). Youden’s index combines sensitivity and specificity into a single metric, ranging from 0 to 1, with higher values indicating better test performance in balancing accurate identification of positive and negative cases [54]. All tools were run using 4 threads for consistency. Runtime and peak memory were calculated as the mean and maximum, respectively, from executing each method 10 times. In the context of this analysis, we define a true positive as a read that originates from a human genome and was classified as being a human read, a true negative as a nonhuman read that was not classified as human, a false positive as a nonhuman read that was classified as a human read, and a false negative as a human read that was not classified as human.

### *Mycobacterium* read classification

Two classification tools, kraken and minimap2, were evaluated with 3 different databases each. For kraken, we used the standard database [55], which contains complete RefSeq genomes from bacterial, archaeal, and viral domains, the human genome, and a collection of known vectors, along with a version of this database that is capped at 8 GB (standard-8) [56]. These 2 databases were built on 5 June 2023. The third kraken database used was a *Mycobacterium*-specific one. To generate this database, we used genome_updater (v0.6.3) [57] to download 1 RefSeq genome from each species in the *Mycobacteriaceae* family, plus 1 RefSeq genome from each species in the following genera: *Klebsiella, Escherichia, Salmonella, Enterobacter, Streptococcus, Staphylococcus, Pseudomonas, Xanthomonas*, and *Bifidobacterium*. We ensured that the genomes used to simulate NTM and MTBC reads were not included in this database. We then built the kraken database from these genomes using kraken2-build --build with default options.

For minimap2, 1 database (MTB) contains the *M. tuberculosis* H37Rv reference genome (RefSeq accession GCF_000195955.2), plus 17 high-quality *M. tuberculosis* references from lineages 1 to 6 [31]. The second database (Clockwork) contains common sputum contaminants along with a selection of NTM genomes and H37Rv—as used in the Clockwork pipeline [14]. In addition, we added the 17 high-quality *M. tuberculosis* genomes to this collection. The third minimap2 database we built was a *Mycobacterium*-specific one. For this database, we used genome_updater to download 1 RefSeq genome from each leaf node in the *Mycobacterium* genus [40, 41] plus the 17 high-quality *M. tuberculosis* assemblies.

The nonhuman reads were used for classification. We ran minimap2 with the present option -x sr for Illumina and -x map-ont for Nanopore along with base-level alignment and no secondary alignments (-c --secondary=no). We use default options for kraken classification, except for the --paired option for Illumina data.

We assess the performance of these tools and databases with the same metrics and threads as human read removal (see “Human read removal”). In the context of this analysis, we define a true positive as a read that originates from a *M. tuberculosis* genome and was classified as being a *M. tuberculosis* read, a true negative as a non–*M. tuberculosis* read that was not classified as *M. tuberculosis*, a false positive as a non–*M. tuberculosis* read that was classified as a *M. tuberculosis* read, and a false negative as a *M. tuberculosis* read that was not classified as *M. tuberculosis*.

### *M. tuberculosis* coverage

We calculate the breadth and depth of coverage of *M. tuberculosis* reads before and after the classification process. To get the preclassification (expected) coverage, we align the true *M. tuberculosis* reads to the H37Rv genome with minimap2 and calculate coverage from that alignment using the samtools (v1.19) command coverage [58]. We then calculate the postclassification depth in the same way, using only the reads classified as *M. tuberculosis*.

## Availability of Source Code and Requirements

All code to perform the analysis in this work and produce the custom databases can be found at the GitHub project described below or WorkflowHub [59]. All steps in the pipeline were executed in reproducible remote containers or conda environments, which are listed in the project configuration file within the repository.

- Project name: Classification benchmark
- Project homepage: https://github.com/mbhall88/classification_benchmark
- Operating system(s): Platform independent
- Programming language: Snakemake [60], Python, Perl, and Bash
- License: MIT

This repository also contains example usage of the custom databases.

## Supplementary Material

giae010_GIGA-D-23-00280_Original_Submission

giae010_GIGA-D-23-00280_Revision_1

giae010_GIGA-D-23-00280_Revision_2

giae010_Response_to_Reviewer_Comments_Original_Submission

giae010_Response_to_Reviewer_Comments_Revision_1

giae010_Reviewer_1_Report_Original_Submission Ben Langmead -- 10/29/2023 Reviewed

giae010_Reviewer_1_Report_Revision_1 Ben Langmead -- 1/17/2024 Reviewed

giae010_Reviewer_2_Report_Original_Submission Darrin Lemmer, M.S. -- 11/1/2023 Reviewed

giae010_Reviewer_2_Report_Revision_1 Darrin Lemmer, M.S. -- 1/30/2024 Reviewed

giae010_Supplemental_Files

## Data Availability

The datasets supporting the results of this study are available in Zenodo and have been cited in Results where first mentioned. They include the simulated Nanopore [17] and Illumina [18] reads, the artificial real Nanopore and Illumina reads [19], the human-only [21] and HPRC [23] kraken databases, the *Mycobacterium*-specific kraken database [30], and the Clockwork [34], *M. tuberculosis–*specific [32], and *Mycobacterium*-specific databases [33] used by minimap2. The standard [55] and standard-8 [56] kraken databases were downloaded from the URLs listed in their respective references. Snapshots of our code and other data further supporting this work are openly available in the *GigaScience* repository, GigaDB [61].

## Additional Files

**Supplementary Table S1**. Performance of human read classification—simulated Nanopore. A more detailed version of Table 1.

**Supplementary Table S2**. Performance of human read classification—real Nanopore. A more detailed version of Table 2.

**Supplementary Table S3**. Performance of human read classification—simulated Illumina. A more detailed version of Table 3.

**Supplementary Table S4**. Performance of human read classification—real Illumina. A more detailed version of Table 4.

**Supplementary Table S5**. Performance of *M. tuberculosis* classification—simulated Nanopore. A more detailed version of Table 5.

**Supplementary Table S6**. Performance of *M. tuberculosis* classification—real Nanopore. A more detailed version of Table 6.

**Supplementary Table S7**. Performance of *M. tuberculosis* classification—simulated Illumina. A more detailed version of Table 7.

**Supplementary Table S8**. Performance of *M. tuberculosis* classification—real Illumina. A more detailed version of Table 8.

**Supplementary Table S9**. Breadth and depth of coverage of *M. tuberculosis* simulated Nanopore reads before and after classification. rel_covg indicates the relative coverage, calculated as pre_covg divided by post_covg.

**Supplementary Table S10**. Breadth and depth of coverage of *M. tuberculosis* real Nanopore reads before and after classification. rel_covg indicates the relative coverage, calculated as pre_covg divided by post_covg.

**Supplementary Table S11**. Breadth and depth of coverage of *M. tuberculosis* simulated Illumina reads before and after classification. rel_covg indicates the relative coverage, calculated as pre_covg divided by post_covg.

**Supplementary Table S12**. Breadth and depth of coverage of *M. tuberculosis* real Illumina reads before and after classification. rel_covg indicates the relative coverage, calculated as pre_covg divided by post_covg.

## Abbreviations

bp: base pairs; FN: false negative; FP: false positive; Gbp: gigabase pairs; HERV-K: human endogenous retrovirus K; HPRC: Human Pangenome Reference Consortium; HRRT: human read removal tool; kbp: kilobase pairs; Mbp: megabase pairs; MTB: *Mycobacterium tuberculosis*; MTBC: *Mycobacterium tuberculosis* complex; NTM: nontuberculous mycobacteria; TP: true positive; WGS: whole-genome sequencing.
