# Supplementary material for: Pangenome databases improve host removal and mycobacteria classification from clinical metagenomic data
Source: Gigascience. 2024 Apr 4;13:giae010. doi: 10.1093/gigascience/giae010 (PMC10993716; doi:10.1093/gigascience/giae010)
Supplement: giae010_Response_to_Reviewer_Comments_Original_Submission [file giae010_response_to_reviewer_comments_original_submission.pdf]

Author's Response To Reviewer Comments

We sincerely thank the reviewers for their detailed and insightful comments. We feel each comment was fair and justified and our manuscript is substantially improved as a result of addressing these questions/issues. A detailed point by point response to each reviewer's comment is provided below, but we highlight a major addition/change here.
Both reviewers requested larger simulations and/or a real dataset and questioned the reliability of the simulations. Firstly, we have now added real Illumina and Nanopore metagenomic datasets. Secondly, we have added confidence intervals to the accuracy metrics and switched from precision (which is prevalence-dependent) to specificity (which is prevalence-independent). We feel these changes and additions greatly improve the reliability and applicability of the results. See the below comments for a detailed explanation of these additions/changes.

Response to comments from editor:

This work does not describe a new software application, but the workflow has now been registered on workflowhub and is cited in the Availability of source code section.

Response to Reviewer 1 comments:

Major Comment 1:
This is a fair point. We have no done three things to address these concerns.
1. We have added evaluation on a real dataset. This is described in the manuscript as an artificial real dataset as we have taken real sequencing reads from samples of a single taxa for human and MTB, and a Zymo mock community metagenomic sample which does not include MTB or human, and combined all of these together into an artificial real metagenomic sample.
2. We have included 95% confidence intervals for all accuracy metrics. This allows one to distinguish whether the results are robust and whether differences between methods are reliable (i.e., do their confidence intervals overlap).
3. We have switched from precision to specificity and retained recall (renaming it as sensitivity). In contrast to precision, specificity (and sensitivity) is prevalence-independent, meaning these results should hold for different metagenomic composition ratios, thus increasing the reliability of the results.

Major Comment 2:
This is a fair point. In our updated results we now use the true non-human reads as the input for the Mycobacterium experiment for both the simulated and real datasets, as requested.

Major Comment 3:
We have included a paragraph near the end of the discussion addressing this.
"Although the read classification and custom databases were targeted at M. tuberculosis, this work should act as a guide for how to design such taxon-specific databases for other species. We advise beginning with simulations of metagenomic samples and determining how much diversity, and which rank, is required from your taxa-of-interest based on sensitivity results. In addition, false positive classifications are a good guide for which taxa outside your species-of-interest may need to be included as a kind of bait."

Major Comment 4:
Of the four alignments of Mycobacterium reads to the human genome in the simulated data, two map to chromosome 6 in gene HSPA1B, but these have mapping quality 0 and have per-base approximate sequence divergence (dv:f) less than 0.16. One maps to chromosome 16 in gene ZFHX3 with mapping quality 7 and sequence divergence 0.12. The last one maps to chromosome 14 in gene HSPA2 with sequence divergence 0.19.

On the simulated data, indeed, Hostile had 4 total FPs (3 Mycobacterium and 1 Tsukamurella) compared to minimap2's 7 FPs (4 Mycobacterium, 1 Halmonas, 1 Tsukamurella and 1 Bovine retro virus), however, on the new real dataset, Hostile had 1274 FPs compared to minimap2's 1380. We have also clarified in the manuscript that the difference between Hostile and our minimap2 approach is not filtering, but base-level alignment. This is the added text:
"It should be noted that while Hostile is using minimap2 and aligning to the same reference genome as the minimap2 configuration, it uses base-level alignment to a SAM file (-a), while the minimap2 configuration uses approximate mapping (default) which is generally less accurate but faster".
So given these differences in alignment approach, we would expect some dissimilarities in the number of FPs between the two approaches.

Major Comment 5:
We have now included the error rates in the methods section. They are: Nanopore median error rate 6.3% and Illumina error rate 0.1%.

Minor Comment 1:
This was predominantly due to kraken not being able to place the missed reads (FNs) at the species rank. In all cases, at least 90% of the FNs were correctly classified at the genus level, with the remainder not being placed even at the genus level. We have added a paragraph about this to the results section.

Minor Comment 2:
Good point. GB units are now used throughout the paper.

Minor Comment 3:
We have added a short analysis to the manuscript showcasing that these scaffolds are not the result of contamination. Here is a summary:
The KOREF genome was split into 100bp overlapping pseudo-reads every 50bp using the SeqKit (v2.4.0) command sliding. The pseudo-reads were then classified with kraken using the standard database. Of the 57943303 pseudo-reads, only 56 were classified as bacteria, with no one family being over-represented. As such, we can conclude these unplaced scaffolds are unlikely to be the result of contamination.

Response to Reviewer 2 comments:

Comment 1:
We agree to a certain extent with this comment and have made the following changes, which we believe address your concerns:
1. We have added evaluation on a real dataset. This is described in the manuscript as an artificial real dataset as we have taken real sequencing reads from samples of a single taxa for human and MTB, and a Zymo mock community metagenomic sample which does not include MTB or human, and combined all of these together into an artificial real metagenomic sample.
2. We have included 95% confidence intervals for all accuracy metrics. This allows one to distinguish whether the results are robust and whether differences between methods are reliable (i.e., do their confidence intervals overlap).
3. We have switched from precision to specificity and retained recall (renaming it as sensitivity). In contrast to precision, specificity (and sensitivity) is prevalence-independent, meaning these results will not change for different metagenomic composition ratios, thus increasing the reliability of the results.
As we are doing read-level-based classification, adding extra simulations would be equivalent to creating a larger single simulation. However, with the addition of confidence intervals, the switch to specificity instead of precision (making the result prevalence independent), and the addition of a real dataset, we feel the findings are now far more robust to different compositions and depth of coverage as well as the standard variability seen in the real world.

Comment 2:
Your point about the results being fairly indistinguishable between the human read removal methods is fair, even with the addition of real data we don't see huge differences in sensitivity and specificity (except for

HRRT). We have added the following sentences to the discussion to highlight this observation: “Another point of note is that while the sensitivity and specificity confidence intervals were often non-overlapping for the best performing methods, with the except of HRRT, they were generally within <1% of other approaches. Therefore, we would not suggest changing existing pipelines that use any of these methods (aside from HRRT), unless improved computational performance is needed.”
We also agree that the word ‘superior’ in the title is perhaps a little strong (though the MTB results are indeed quite a bit better) and have changed the title to “Pangenome databases improve host removal and mycobacteria classification from clinical metagenomic data”.
Regarding the run time and peak memory usage, we have now changed these to the mean run time and maximum memory usage from 10 executions.

Comment 3:
We hope that the previous comments have addressed the issues raised in this comment around multiple datasets and multiple runs of each method.
Regarding the breadth and depth of coverage, we have included a new analysis addressing this (see Methods, Results and Discussion). Indeed, there is not much reduction in either coverage, as is expected due to the high sensitivity of most methods. However, the standard kraken databases provide an enlightening result that highlights some benefits of long-read Nanopore data over short Illumina.

Comment 4:
As mentioned in a previous comment, we have now added analysis of an artificial real dataset and we hope this satisfies your comment. Our reasoning for creating such a dataset were two-fold. Reference #8 (which MH is a co-first-author) did not, and cannot upload the human component of these datasets, as with nearly any MTB sputum sample, due to ethical and privacy reasons. And even if we could get such a real dataset, we would not know the taxonomic truth of any read, as it is a true metagenomic mixture. Therefore, we decided to create our dataset by combining real samples where we are highly confident of the species contained within.
