# Supplementary material for: Pangenome databases improve host removal and mycobacteria classification from clinical metagenomic data
Source: Gigascience. 2024 Apr 4;13:giae010. doi: 10.1093/gigascience/giae010 (PMC10993716; doi:10.1093/gigascience/giae010)
Supplement: giae010_Response_to_Reviewer_Comments_Revision_1 [file giae010_response_to_reviewer_comments_revision_1.pdf]

Author's Response To Reviewer Comments

I have added the minimap2 and kraken RRIDs, but instead of adding them where the annotation was in the PDF, I added them on the first mention of the tool - I hope this is preferable.

I have removed the requested URLs from the text, but did not add them to the reference list as the items were already referenced and the entry in the bibliography already has the appropriate URL. So the URLs I had added in the text was redundant anyway.

I have added the requested text and citation for the GigaDB record.
