# Supplementary material for: Pangenome databases improve host removal and mycobacteria classification from clinical metagenomic data
Source: Gigascience. 2024 Apr 4;13:giae010. doi: 10.1093/gigascience/giae010 (PMC10993716; doi:10.1093/gigascience/giae010)
Supplement: giae010_Reviewer_1_Report_Original_Submission — Ben Langmead -- 10/29/2023 Reviewed [file giae010_reviewer_1_report_original_submission.pdf]

Reviewer Report

Title: Pangenome databases improve host removal and mycobacteria classification from clinical metagenomic data

Version: Original Submission **Date:** 10/29/2023

Reviewer name: Ben Langmead

Reviewer Comments to Author:

Mycobacterium tuberculosis is a leading cause of death and is increasingly investigated using DNA sequencing of e.g. sputum. However sequencing data has to be handled carefully to remove reads from the human host and to correct classify the M. tuberculosis reads.
The authors focus on this problem of computationally extracting only the M. tuberculosis reads from a simulated human sample, measuring the accuracy and time/memory resources used by different approaches. They begin by focusing on removing human reads using different alignment and k-mer-based tools. The authors discover, interestingly, that using a Kraken database over all the HPRC genomes leads to the best balance of resources and accuracy. Next, the authors focus on classifying the remaining reads with various databases, some general across the tree-of-life and some limited to M. tuberculosis. For this task, the authors identify the custom Mycobacterium databases as being the best choice to correctly identify
tuberculosis efficiently.
The paper is very clear and well written.
Major Comments:
1. While the host-depletion and mycobacterium classification do tell us something, some of the numbers are quite small, leading me to wonder how robust the results are. The question lingers: should we really be making dedicions based on a simulation study where results are similar out to the fifth decimal point? There is definitely information here, but additionally evaluating real datasets or still-larger simulated ones could make the results more actionable.
2. In the Mycobacterium experiment, it does not seem approriate to use the reads not classified by Kraken as the inputs, since Kraken is what is being benchmarked in the first place. Given this is a simulation data, an alternative would be to use true non-human reads as the input.
3. The Discussion could be improved with some discussion of whether these approaches could generalize to other taxa, or other host/pathogen combinations.
4. In Table 3, the authors point out that minimap2 is the only tool to misclassify Mycobacterium reads as Human. Relatively, it has the most FPs compared to the other tools. Did the authors investigate where those alignments fell within CHM13v2? it's mentioned that Hostile uses minimap2 but with extra filtering, so I'm surprised it only has 4 FPs. Does that make sense given the specific filtering steps it performs after alignment?
5. I may have missed it, but the authors should characterize the error rate for the simulated ONT and Illumina reads somewhere. Saying the "default model" is used doesn't help the reader to understand the error profile.

Minor Comments:
1. Kraken has substantially low recall using the standard and standard-8 databases in Tables 4 and 5. Those were the only times a tool had a recall below 97%. Is this expected? Perhaps because key strains are missing from the database? This wasn't explained.
2. The units of the peak memory in the tables are in MB, but memory thresholds are described using GB units in the paper. Consider changing tables to be in GB.
3. At the end of the host-depletion section, it's mentioned that all missed human reads (FNs) were from unplaced scaffolds. Is it known if those matches are due to contamination in the assembly? Contigs under 10 kb were filtered, but there could be contaminated contigs above that length.

Methods

Are the methods appropriate to the aims of the study, are they well described, and are necessary controls included? Choose an item.

Conclusions

Are the conclusions adequately supported by the data shown? Choose an item.

Reporting Standards

Does the manuscript adhere to the journal's guidelines on minimum standards of reporting? Choose an item.

Choose an item.

Statistics

Are you able to assess all statistics in the manuscript, including the appropriateness of statistical tests used? Choose an item.

Quality of Written English

Please indicate the quality of language in the manuscript: Choose an item.

Declaration of Competing Interests

Please complete a declaration of competing interests, considering the following questions:

- Have you in the past five years received reimbursements, fees, funding, or salary from an organisation that may in any way gain or lose financially from the publication of this manuscript, either now or in the future?
- Do you hold any stocks or shares in an organisation that may in any way gain or lose financially from the publication of this manuscript, either now or in the future?

- Do you hold or are you currently applying for any patents relating to the content of the manuscript?
- Have you received reimbursements, fees, funding, or salary from an organization that holds or has applied for patents relating to the content of the manuscript?
- Do you have any other financial competing interests?
- Do you have any non-financial competing interests in relation to this paper?

If you can answer no to all of the above, write 'I declare that I have no competing interests' below. If your reply is yes to any, please give details below.

I declare that I have no competing interests

I agree to the open peer review policy of the journal. I understand that my name will be included on my report to the authors and, if the manuscript is accepted for publication, my named report including any attachments I upload will be posted on the website along with the authors' responses. I agree for my report to be made available under an Open Access Creative Commons CC-BY license (http://creativecommons.org/licenses/by/4.0/). I understand that any comments which I do not wish to be included in my named report can be included as confidential comments to the editors, which will not be published.

Choose an item.

To further support our reviewers, we have joined with Publons, where you can gain additional credit to further highlight your hard work (see: https://publons.com/journal/530/gigascience). On publication of this paper, your review will be automatically added to Publons, you can then choose whether or not to claim your Publons credit. I understand this statement.

Yes Choose an item.
