# Supplementary material for: Pangenome databases improve host removal and mycobacteria classification from clinical metagenomic data
Source: Gigascience. 2024 Apr 4;13:giae010. doi: 10.1093/gigascience/giae010 (PMC10993716; doi:10.1093/gigascience/giae010)
Supplement: giae010_Reviewer_2_Report_Original_Submission — Darrin Lemmer, M.S. -- 11/1/2023 Reviewed [file giae010_reviewer_2_report_original_submission.pdf]

Reviewer Report

Title: Pangenome databases improve host removal and mycobacteria classification from clinical metagenomic data

Version: Original Submission **Date:** 11/1/2023

Reviewer name: Darrin Lemmer, M.S.

Reviewer Comments to Author:

This paper describes a method for improving the accuracy and efficiency of extracting a pathogen of interest (M. tuberculosis in this instance, though the methods should work equally well for other pathogens) from a "clinical" metagenomic sample. The paper is well written and provides links to all source code and datasets used, which were well organized and easy to understand. The premise -- that using a pangenome database improves classification -- seems pretty intuitive, but it is nice to see some benchmarking to prove it. For clarity I will arrange my comments by the three major steps of your methods: dataset generation, human read removal, and Mycobacterium read classification.

1. Dataset generation -- I appreciate that you used a real-world study (reference #8) to approximate the proportions of organisms in your sample, however I am disappointed that you generated exactly one dataset for benchmarking. Even if you use the exact same community composition, there is a level of randomness involved in generating sequencing reads, and therefore some variance. I would expect to see multiple generations and an averaging of the results in the tables, however with a sufficiently high read depth, the variance won't likely change your results much, so it would be nice, and more true to real sequencing data, to vary the number of reads generated (I didn't see where you specified to what read depth for each species you generated the reads for), as it is rare in the real world to always get this deep of coverage. Ideally it would also be nice to see datasets varying the proportions of MTBC in the sample to test the limits of detection, but that may be beyond the scope of this particular paper.

2. Human read removal -- The data provided do not really support the conclusion, as all methods benchmarked performed quite well and, particularly when using the long reads from the Nanopore simulated dataset, fairly indistinguishable with the exception of HRRT. The short Illumina reads show a little more separation between the methods, probably due to the shorter sequences being able to align to multiple sequences in the reference databases, however comparing kraken human to kraken HPRC still shows very little difference, thus not supporting the conclusion that the pangenome reference provides "superior" host removal. The run times and memory used do much more to separate the performance of the various methods, and particularly with the goal of being able to run the analysis on a personal computer where peak memory usage is important. The only methods that perform well within the memory constraints of a personal computer for both long reads and short leads are HRRT and the two kraken methods, with kraken being superior at recall, but again, kraken human and kraken HPRC are virtually indistinguishable, making it hard to justify the claim that the pangenome is superior. Also, it appears your run time and peak memory usage is again based on one single data point, these should be performed multiple times and averaged. Finally, as an aside, I did find it interesting and disturbing that HRRT had such a high false negative rate compared to the other methods, given that this is the primary

method used by NCBI for publishing in the SRA database, implying there are quite a few human remaining in SRA.
3. Mycobacterium read classification -- Here we do have some pretty good support for using a pangenome reference database, particularly compared to the kraken standard databases, though as mentioned previously, a single datapoint isn't really adequate, and I'd like to see both multiple datasets and multiple runs of each method. Additionally, given the purpose here is to improve the amount of MTB extracted from a metagenomic sample, these data should be taken the one extra step to show the coverage breadth and depth of the MTB genome provided by the reads classified as MTB, as a high number of reads doesn't mean much if they are all stacked at the same region of the genome. Given that these are simulated reads, which tend to have pretty even genome coverage, this may not show much, however it is still an important piece to show the value of your recommended method.
One final comment is that it should be fairly easy to take this beyond a theoretical exercise, by running some actual real world datasets through the methods you are recommending to see how well they perform in actuality. For instance, reference #8, which you used as a basis for the composition of your simulated metagenomic sample, published their actual sequenced sputum samples. It would be easy to show if you can improve the amount of Mycobacterium extracted from their samples over the methods they used, thus showing value to those lower income/high TB burden regions where whole meta genome sequencing may be the best option they have.

Methods

Are the methods appropriate to the aims of the study, are they well described, and are necessary controls included? Choose an item.

Conclusions

Are the conclusions adequately supported by the data shown? Choose an item.

Reporting Standards

Does the manuscript adhere to the journal's guidelines on minimum standards of reporting? Choose an item.

Choose an item.

Statistics

Are you able to assess all statistics in the manuscript, including the appropriateness of statistical tests used? Choose an item.

Quality of Written English

Please indicate the quality of language in the manuscript: Choose an item.

Declaration of Competing Interests

Please complete a declaration of competing interests, considering the following questions:

- Have you in the past five years received reimbursements, fees, funding, or salary from an organisation that may in any way gain or lose financially from the publication of this manuscript, either now or in the future?
- Do you hold any stocks or shares in an organisation that may in any way gain or lose financially from the publication of this manuscript, either now or in the future?
- Do you hold or are you currently applying for any patents relating to the content of the manuscript?
- Have you received reimbursements, fees, funding, or salary from an organization that holds or has applied for patents relating to the content of the manuscript?
- Do you have any other financial competing interests?
- Do you have any non-financial competing interests in relation to this paper?

If you can answer no to all of the above, write 'I declare that I have no competing interests' below. If your reply is yes to any, please give details below.

I declare that I have no competing interests

I agree to the open peer review policy of the journal. I understand that my name will be included on my report to the authors and, if the manuscript is accepted for publication, my named report including any attachments I upload will be posted on the website along with the authors' responses. I agree for my report to be made available under an Open Access Creative Commons CC-BY license (http://creativecommons.org/licenses/by/4.0/). I understand that any comments which I do not wish to be included in my named report can be included as confidential comments to the editors, which will not be published.

Choose an item.

To further support our reviewers, we have joined with Publons, where you can gain additional credit to further highlight your hard work (see: https://publons.com/journal/530/gigascience). On publication of this paper, your review will be automatically added to Publons, you can then choose whether or not to claim your Publons credit. I understand this statement.

Yes Choose an item.
