# Supplementary material for: Pangenome databases improve host removal and mycobacteria classification from clinical metagenomic data
Source: Gigascience. 2024 Apr 4;13:giae010. doi: 10.1093/gigascience/giae010 (PMC10993716; doi:10.1093/gigascience/giae010)
Supplement: giae010_Reviewer_2_Report_Revision_1 — Darrin Lemmer, M.S. -- 1/30/2024 Reviewed [file giae010_reviewer_2_report_revision_1.pdf]

Reviewer Report

Title: Pangenome databases improve host removal and mycobacteria classification from clinical metagenomic data

Version: Revision 1 **Date:** 1/30/2024

Reviewer name: Darrin Lemmer, M.S.

Reviewer Comments to Author:

This is a significantly stronger paper than originally submitted. I especially appreciate that multiple runs have now been done with more than one dataset, including a "real" dataset, and the analysis showing the breadth and depth of coverage of the retained Mtb reads, proving that you can still generally get a complete genome of a metagenomic sample with these methods. However kraken's low sensitivity when using the standard database definitely impacts the results, making a stronger argument for using a pangenome database (Kraken-Standard can identify the presence of Mtb, but if you want to do anything more with it, like AMR detection, you would need to use a pangenome database). I really think that this should be emphasized more, and perhaps some or all of the data in tables S9-S12 be brought into the main paper. It is maybe worth noting, that the significant drop in breadth, I would imagine, is a result of dividing the total size of the aligned reads by the size of the genome, implying a shallow coverage, but the reality is still high coverage in the areas that are covered, but lots of complete gaps in coverage. I did also like the switch to the somewhat more standard sensitivity/specificity metrics, though I do lament the actual FN/FP counts being relegated to the supplemental tables, as I thought these numbers valuable (or at least interesting) when comparing the results of the various pipelines, particularly with human read removal, where the various pipelines perform quite similarly.

Methods

Are the methods appropriate to the aims of the study, are they well described, and are necessary controls included? Choose an item.

Conclusions

Are the conclusions adequately supported by the data shown? Choose an item.

Reporting Standards

Does the manuscript adhere to the journal's guidelines on minimum standards of reporting? Choose an item.

Choose an item.

Statistics

Are you able to assess all statistics in the manuscript, including the appropriateness of statistical tests used? Choose an item.

Quality of Written English

Please indicate the quality of language in the manuscript: Choose an item.

Declaration of Competing Interests

Please complete a declaration of competing interests, considering the following questions:

- Have you in the past five years received reimbursements, fees, funding, or salary from an organisation that may in any way gain or lose financially from the publication of this manuscript, either now or in the future?
- Do you hold any stocks or shares in an organisation that may in any way gain or lose financially from the publication of this manuscript, either now or in the future?
- Do you hold or are you currently applying for any patents relating to the content of the manuscript?
- Have you received reimbursements, fees, funding, or salary from an organization that holds or has applied for patents relating to the content of the manuscript?
- Do you have any other financial competing interests?
- Do you have any non-financial competing interests in relation to this paper?

If you can answer no to all of the above, write 'I declare that I have no competing interests' below. If your reply is yes to any, please give details below.

I declare that I have no competing interests

I agree to the open peer review policy of the journal. I understand that my name will be included on my report to the authors and, if the manuscript is accepted for publication, my named report including any attachments I upload will be posted on the website along with the authors' responses. I agree for my report to be made available under an Open Access Creative Commons CC-BY license (http://creativecommons.org/licenses/by/4.0/). I understand that any comments which I do not wish to be included in my named report can be included as confidential comments to the editors, which will not be published.

Choose an item.

To further support our reviewers, we have joined with Publons, where you can gain additional credit to further highlight your hard work (see: https://publons.com/journal/530/gigascience). On publication of this paper, your review will be automatically added to Publons, you can then choose whether or not to claim your Publons credit. I understand this statement.

Yes Choose an item.
